# 肺癌预防性脑照射的研究进展

**DOI:** 10.3779/j.issn.1009-3419.2012.09.08

**Published:** 2012-09-20

**Affiliations:** 1 310053 杭州，浙江中医药大学第二临床医学院 The Second Clinical Medical College, Zhejiang Chinese Medical University, Hangzhou 310053, China; 2 310006 杭州，杭州市第一人民医院医疗集团，杭州市肿瘤医院放疗科 Department of Radiation Oncology, Hangzhou First People's Hospital, Hangzhou Cancer Hospital, Hangzhou 310006, China

肺癌患者经过治疗后，面临三种治疗失败的潜在风险：局部复发、脑外器官转移和脑转移。随着多元化诊治手段的发展，脑转移日渐成为影响患者预后的关键因素。发生脑转移后全脑放疗中位生存期仅为4个月-6个月^[1, 2]^，且患者生活质量受到严重影响。因此，如何有效预测及预防肺癌脑转移的发生，有选择地实施预防性脑照射（prophylactic cranial irradiation, PCI）日益成为临床关注的热点。

## 1 PCI用于小细胞肺癌（small cell lung cancer, SCLC）

SCLC生物学行为激进，首诊时约15%的病例合并脑转移^[3]^，局限期SCLC治疗后达完全缓解的患者约50%会出现脑转移^[4]^，因此PCI的研究首先在SCLC中开展。

### 1.1 PCI在局限期SCLC中的应用

Aupérin等^[5]^对7项临床随机对照试验进行了荟萃分析，共入组987例完全缓解的SCLC患者，其中85%为局限期SCLC患者，15%为广泛期SCLC患者。分析发现PCI治疗组较对照组脑转移率降低了25%（*P* < 0.001），3年生存率提高了5.4%（*P*=0.01），由此确立了PCI在局限期SCLC治疗中的地位。但是近1/3的患者接受PCI治疗后仍然发生脑转移，这部分患者对二程放疗的有效率不到50%，预计生存期为4个月-6个月^[6]^。该荟萃分析按全脑照射剂量8 Gy、24 Gy-25 Gy、30 Gy以及36 Gy-40 Gy将接受PCI的患者分为4组，发现脑转移发生风险分别会降低24%、48%、66%和73%，提示需要进一步摸索PCI的最佳治疗剂量以降低脑转移发生率并考虑到可能的神经系统损害。

Le Péchoux等^[7]^在2009年公布了一项随机临床研究（PCI 01-EULINT1），比较了高剂量和低剂量PCI在预防脑转移发生中的作用。该试验将720例获得完全缓解的局限期SCLC患者随机分入低剂量组（25 Gy/10 f）和高剂量组（36 Gy），高剂量组又分为两种剂量分割模式：常规分割（36 Gy/18 f/18 d）和加速超分割（36 Gy/24 f/16 d）。主要研究终点是2年的脑转移发生率，次要研究终点为生存期、生活质量和迟发后遗症的评定。结果显示2年累积脑转移发生率以高剂量组为优（16% *vs* 22%, *P*=0.005）。令人意外的是，PCI高剂量组出现了更多的胸内疾病进展（48% *vs* 40%, *P*=0.02），2年生存率低于PCI低剂量组（37% *vs* 42%）。然而不能简单凭上述结果就理解为高剂量PCI无法为患者带来生存的获益，该研究设计的首要终点指标为2年脑转移发生率，尚缺乏足够的统计数据检测出生存上的优劣，两组生存上差异可能是一个假阳性的结果。另外，尽管该试验提供的两组患者基线特征分布均衡，但未提供胸部放疗剂量的分割、时机等，考虑到该研究纳入了包括22个国家157个研究中心的患者，在胸部放射治疗的实施中可能存在较大的差异，PCI高剂量组中较差的胸内疾病控制以及较差的预后可能与此有关。肿瘤放射治疗协作组织（Radiation Therapy Oncology Group, RTOG）0212^[8]^是PCI 01-EULINT1试验的参与组之一，它的研究目的是评价高PCI剂量对神经系统的毒性和对生活质量的影响。该试验发现虽然不同剂量PCI组患者包括NPTB的4项指标和生活质量的2项量表无统计学差异，但低剂量组（总照射剂量25 Gy）发生神经系统毒性的风险明显低于高剂量组（总照射剂量36 Gy，*P*=0.02），因此目前高剂量不作为常规推荐。

既往研究认为对于获得完全缓解的局限期SCLC患者应在化疗结束后行PCI。为了探讨不同时间行PCI对脑转移发生率的影响，Sas-Korczyńska等^[9]^对129例同步放化疗后完全缓解的局限期SCLC患者进行了研究，结果表明早期行PCI治疗（胸部放疗结束到最后一个化疗周期之前）较晚期行PCI治疗（放化疗结束后）明显提高4年无脑转移生存率（*P* < 0.001），提示局限期SCLC早期行PCI获益更大。

### 1.2 PCI在广泛期SCLC中的应用

欧洲癌症研究与治疗组织（European Organisation for Research and Treatment of Cancer, EORTC）放射肿瘤和肺癌组在2007年公布的一项随机临床试验^[10]^将入组的286例既往行4周期-6周期化疗后获得缓解的广泛期SCLC患者随机分入PCI组（20 Gy-30 Gy）和对照组，结果显示1年累计脑转移发生降低（14.6% *vs* 40.4%, *P* < 0.001），1年生存率延长（13.3% *vs* 27.1%）。这项研究为广泛期小细胞肺癌的治疗带来了新的治疗策略。

2012年的NCCN指南推荐PCI剂量为25 Gy/10 f或者30 Gy/15 f；对于广泛期SCLC患者，由于预计生存期较短，可酌情给予20 Gy/5 f的剂量。此外，目前已有的证据^[11, 12]^显示化疗后未获得缓解的SCLC患者也可从PCI中获益。

## 2 PCI用于非小细胞肺癌（non-small cell lung cancer, NSCLC）

近年来，随着治疗手段的进步，局部晚期NSCLC的生存率有了一定的延长；而影像学技术的进步也使得更多的脑转移得以明确诊断，脑转移累积发生率为17%-54%，15%-40%的患者以脑转移为首发治疗失败原因^[13, 14]^。脑转移发生率的上升需要更为有效的脑转移治疗方法。PCI在SCLC中运用的成功经验能否在局部晚期NSCLC中得到复制值得深入研究。

### 2.1 PCI用于NSCLC的临床研究

Gaspar等^[13]^分析了4项西南肿瘤组（Southwest Oncology Group, SWOG）肺癌放化疗的临床研究，结果显示71例发生脑转移的局部晚期NSCLC患者中，46%的病例发生在诊断后16周内，83%发生在诊断后1年之内，提示脑转移多出现在疾病初期。Topkan等^[15]^回顾性分析了134例不同方案放化疗后行PCI的局部晚期NSCLC患者，结果表明早期行PCI脑转移率明显降低（*P*=0.03），无脑转移生存期明显延长（*P* < 0.001），该研究支持局部晚期NSCLC患者早期行PCI治疗。

PCI用于NSCLC的5项非随机临床研究^[16-20]^显示PCI可使颅外疾病控制的局部晚期NSCLC患者获益（表 1）。其中Stuschke等^[20]^报道了75例IIIa/IIIb期患者接受诱导放化疗加手术切除，由于研究期间脑转移发生率较高，在后期研究中加PCI（30 Gy/15 f/3 w），研究结果表明PCI降低了4年内脑转移为首发失败的发生率（30% *vs* 8%），且总的脑转移率也从54%降至13%（*P* < 0.001）。

**1:** PCI用于局部晚期NSCLC的非随机临床研究 Nonrandomized studies on PCI in local advanced NSCLC

Reference	*n*	Histology	Primary Tx	Dose of PCI (Gy)	BM		*P*
					PCI (–)	PCI (+)	
Albain *et al*^[16]^	126	All NSCLC	Trimodality	36 (2 Gy/fr)	16%	8%	0.36
Strauss *et al* ^[17]^	41	Non-epidermoid	Trimodality	30 (2 Gy/fr)	12%	0	0.32
Skarin *et al*^[18]^	41	All NSCLC	Trimodality	NS	27%	14%	-
Rusch *et al*^[19]^	73	NS	Ctx+RT	36 (2 Gy/fr)	-	0/73%	-
				30(3 Gy/fr)			
Stuschke *et al* ^[20]^	75	All NSCLC	Trimodality	30 (2 Gy/fr)	54%	13%	< 0.01
Tx: treatment; PCI: prophylactic cranial irradiation; BM: brain metastases; NSCLC: non small cell lung cancer; fr: fraction; NS: not specified; Ctx: chemotherapy; RT: radiation treatment.								

早期报道的PCI用于NSCLC的随机临床研究^[21-26]^结果大体一致（表 2），PCI能降低NSCLC患者脑转移发生率，但不能延长总生存期（overall survival, OS）。近期开展的RTOG 0214试验^[26]^在肿瘤治疗手段进步的基础上重新评估了PCI在NSCLC中的价值。该试验入组的IIIa期、IIIb期的患者已完成胸部病灶的根治性治疗（手术或同步放化疗），无局部区域进展或远处转移。患者被随机分入PCI（30 Gy/15 f/3 w）和观察组。该试验计划入组1, 058例患者，最终因入组效率低，于2007年8月提前终止，实际入组356例患者。结果显示1年生存率及无病生存率无统计学差异，而PCI组较对照组发生脑转移的机会明显减少，亦即得出了与之前随机试验相似的结果。

**2:** PCI用于局部晚期NSCLC的随机临床研究 Randomized studies on PCI in local advanced NSCLC

Reference	*n*	Histology	Primary Tx	Dose of PCI, Gy	BM		*P*
					PCI (–)	PCI (+)	
Cox *et al*^[21]^	281	All NSCLC	RT	20 (2 Gy/fr)	13%	6%	0.04
Umsawasdi *et al*^[22]^	97	All NSCLC	Trimodality	30 (3 Gy/fr)	27%	4%	< 0.01
			Ctx+RT				
Russell *et al*^[23]^	187	Non-epidermoid	RT	30 (3 Gy/fr)	19%	9%	0.10
Mira *et al*^[24]^	229	All NSCLC	Ctx+RT	30 (2 Gy/fr)	11%	0	< 0.01
				37.5 (2.5Gy/fr)			
Pöttgen *et al*^[25]^	112	All NSCLC	Trimodality	30 (2 Gy/fr)	34.7% (FSF)	7.8 % (FSF)	0.02
		(All stage Ⅲa)			27.2%	9.1%	0.04
Gore *et al*^[26]^	340	All NSCLC (All stage Ⅲ)	RT/S	30 (2Gy/fr)	18%	7.7%	< 0.01
FSF: first site of failure; S: surgury.								

吴一龙等^[27]^开展的一项随机多中心Ⅲ期临床研究对局部晚期NSCLC患者化疗后PCI进行了探讨。该研究将仅接受过3周期-6周期化疗并且有临床获益包括疾病稳定和疾病缓解的晚期NSCLC患者随机分入PCI组（30 Gy/10 f）和对照组，主要终点是脑转移发生率，研究正在进行中。从目前的结果来看PCI可降低或延迟化疗后晚期NSCLC患者脑转移的发生率，但对OS是否有益尚不得而知。另一项研究是Komaki等^[28]^对可切除的肺上沟瘤综合治疗的研究，旨在评估治疗副反应、局部控制率以及局部复发远处转移易发的部位及发生时间，其结果拭目以待。

### 2.2 NSCLC发生脑转移的高危人群

PCI可以降低局部晚期NSCLC脑转移的发生率，但不能提高OS，同时可能造成神经系统损害。基于此，许多学者致力于寻找NSCLC脑转移的高危因素、脑转移发生风险预测分子标志物，以便筛选出可以从PCI中获益的发生脑转移的高危人群。从以往的临床研究来看，脑转移的易发因素可能包括以下几项：腺癌（脑转移的发生率为24%-36%^[29]^），年轻^[30]^，女性^[31]^，T、N分期高^[32]^等。此外，利用分子标记物预测脑转移是目前热门的研究方向。D’Amico等^[33]^回顾分析了202例Ⅰ期NSCLC的手术标本，其中25例最终发生了孤立性脑转移。在这项研究中，所有标本都进行了8个分子标记物的检测，分别是erbB2、p53、factor Ⅷ、EphA2和E-cadherin、UPA、UPAR和PAI-1。结果显示P53、UPA高表达与脑转移的发生相关，E-cadherin表达者脑转移发生率降低。而Bubb等^[34]^比较了29例肺癌合并脑转移与29例配对无脑转移肺癌病理标本，检测Ki-67、p53和bcl-2的表达情况，原发肿瘤和脑转移肿瘤的检测结果高度相关，但3个标志物对脑转移发生均无预测能力，在此领域还需要更多深入的研究。

## 3 PCI对神经认知功能的影响

Arriagada等^[4]^以神经心理学测试及脑CT在治疗前以及治疗后6个月、18个月、30个月、48个月对患者进行评价，发现PCI组与对照组相比，神经系统功能受损的发生概率、CT显示脑萎缩和脑室扩张的发生率均无明显差异。相似的结果见于Gregor等^[35]^报道的314例SCLC患者行PCI的随机对照研究，在PCI前、治疗6个月和1年内均存在神经认知功能及生活质量的降低，但PCI组和观察组之间无统计学差异。Grosshans等^[36]^研究发现SCLC患者神经系统功能损害在行PCI治疗前就已存在，治疗后损害并未持续加重。从目前的研究来看，在20 Gy-30 Gy, 2 Gy-3 Gy/f的PCI剂量下，治疗后1年-2年内不会引起明显的神经认知功能的下降，但长期的副作用仍需进一步研究。

## 4 PCI脑部损伤可能的机制

早期研究^[37]^认为，PCI远期副反应包括大脑认知减退和小脑功能失调。近年有研究发现学习、记忆功能减退是由于放射线对海马体的破坏。Monje等^[38]^发现海马体中干细胞对射线极为敏感，放射性的炎症可以导致微环境的改变而迫使神经元祖细胞分化为胶质细胞造成神经细胞缺失，记忆减退。然而海马体的位置及解剖结构的特殊性对放射线的绕行造成了一定的困扰，新技术的出现为保护海马体带来了希望，Gondi等^[39]^对5例曾接受全脑放疗的患者进行剂量学比较，发现调强放疗与TOMO相比，同样可以在保证靶区剂量的同时做到给予海马体保护。Tarnawski等^[40]^研究也发现调强放疗与TOMO均可以在减少神经干细胞45%放射剂量的同时保证靶区剂量。因此采用精确放疗技术来减少PCI的神经毒性以及改善OS和生活质量是合理可行的。此外，应用药物阻止或者延缓PCI神经毒性的发生也是PCI研究的一个重要内容，正在进行的随机双盲安慰剂对照试验RTOG0614^[41]^旨在研究抗老年痴呆药物美金刚是否能够预防全脑放疗副作用，结果值得期待。

## 5 结语

脑转移已经成为了肺癌患者治疗失败的最主要因素。现有临床研究证实PCI能降低完全缓解/部分缓解的SCLC患者脑转移的发生率，提高OS；但还没有足够的证据支持PCI在局部晚期NSCLC中的应用，临床医生需根据病人情况作出个体化选择。期待未来的研究能在SCLC行PCI的最佳剂量、NSCLC易发生脑转移的高危人群判断以及PCI远期毒性等方面取得进展。

## References

[1] Langer CJ, Mehta MP (2005). Current management of brain metastases, with a focus on systemic options. J Clin Oncol.

[2] Kepka L, Cieslak E, Bujko K (2005). Results of the whole-brain radiotherapy for patients with brain metastases from lung cancer: the RTOG RPA intra-classes analysis. Acta Oncol.

[3] Hochstenbag MM, Twijnstra A, Wilmink JT (2000). Asymptomatic brain metastases (BM) in small cell lung cancer (SCLC): MR-imaging is useful at initial diagnosis. J Neurooncol.

[4] Arriagada R, Le Chevalier T, Borie F (1995). Prophylactic cranial irradiation for patients with small-cell lung cancer in complete remission. J Natl Cancer Inst.

[5] Aupérin A, Arriagada R, Pignon JP (1999). Prophylactic cranial irradiation for patients with small-cell lung cancer in complete remission. Prophylactic Cranial Irradiation Overview Collaborative Group. N Engl J Med.

[6] Castrucci WA, Knisely JP (2008). An update on the treatment of CNS metastases in small cell lung cancer. Cancer J.

[7] Le Péchoux C, Dunant A, Senan S (2009). Standard-dose versus higher-dose prophylactic cranial irradiation (PCI) in patients with limited-stage small-cell lung cancer in complete remission after chemotherapy and thoracic radiotherapy (PCI 99-01, EORTC 22003-08004, RTOG 0212, and IFCT 99-01): a randomised clinical trial. Lancet Oncol.

[8] Wolfson AH, Bae K, Komaki R (2011). Primary analysis of a phase Ⅱ randomized trial Radiation Therapy Oncology Group (RTOG) 0212: impact of different total doses and schedules of prophylactic cranial irradiation on chronic neurotoxicity and quality of life for patients with limited-disease small-cell lung cancer. Int J Radiat Oncol Biol Phys.

[9] Sas-Korczyńska B, Korzeniowski S, Wójcik E (2010). Comparison of the effectiveness of " late" and " early" prophylactic cranial irradiation in patients with limited-stage small cell lung cancer. Strahlenther Onkol.

[10] Slotman B, Faivre-Finn C, Kramer G (2007). Prophylactic cranial irradiation in extensive small-cell lung cancer. N Engl J Med.

[11] Viani GA, Boin AC, Ikeda VY (2012). Thirty years of prophylactic cranial irradiation in patients with small cell lung cancer: a *meta*-analysis of randomized clinical trials. J Bras Pneumol.

[b12] 12Tai P, Assouline A, Joseph K, *et al. * Prophylactic cranial irradiation for patients with limited-stage small-cell lung cancer with response to chemoradiation. Clin Lung Cancer, 2012 Jun 4. [Epub ahead of print]

[13] Gaspar LE, Chansky K, Albain KS (2005). Time from treatment to subsequent diagnosis of brain metastases in stage Ⅲ non-small-cell lung cancer: a retrospective review by the Southwest Oncology Group. J Clin Oncol.

[14] Carolana H, Suna AY, Bezjak A (2005). Does the incidence and outcome of brain metastases in locally advanced non-small cell lung cancer justify prophylactic cranial irradiation or early detection?. Lung Cancer.

[15] Topkan E, Parlak C, Kotek A (2012). Impact of prophylactic cranial irradiation timing on brain relapse pates in patients with stage ⅢB non-small-cell lung carcinoma treated with two different chemoradiotherapy regimens. Int J Radiat Oncol Biol Phys.

[16] Albain KS, Rusch VW, Crowley JJ (1995). Concurrent cisplatin/etoposide plus chest radiotherapy followed by surgery for stages IIIA (N2) and IIIB non-small-cell lung cancer: mature results of Southwest Oncology Group phase Ⅱ study 8805. J Clin Oncol.

[17] Strauss GM, Herndon JE, Sherman DD (1992). Neoadjuvant chemotherapy and radiotherapy followed by surgery in stage ⅢA non-small cell carcinoma of the lung: report of a Cancer and Leukemia Group B phase Ⅱ study. J Clin Oncol.

[18] Skarin A, Jochelson M, Sheldon T (1989). Neoadjuvant chemotherapy in marginally resectable stage Ⅲ M0 non-small cell lung cancer: long-term follow-up in 41 patients. J Surg Oncol.

[19] Rusch VW, Griffin BR, Livingston RB (1989). The role of prophylactic cranial irradiation in regionally advanced non-small cell lung cancer. A Southwest Oncology Group Study. J Thorac Cardiovasc Surg.

[20] Stuschke M, Eberhardt W, Pottgen C (1999). Prophylactic cranial irradiation in locally advanced non-small-cell lung cancer after multimodality treatment: long-term follow-up and investigations of late neuropsychologic effects. J Clin Oncol.

[21] Cox JD, Stanley K, Petrovich Z (1981). Cranial irradiation in cancer of the lung of all cell types. JAMA.

[22] Umsawasdi T, Valdivieso M, Chen TT (1984). Role of elective brain irradiation during combined chemoradiotherapy for limited disease non-small cell lung cancer. J Neurooncol.

[23] Russell AH, Pajak TE, Selim HM (1991). Prophylactic cranial irradiation for lung cancer patients at high risk for development of cerebral metastasis: results of a prospective randomized trial conducted by the Radiation Therapy Oncology Group. Int J Radiat Oncol Biol Phys.

[24] Mira JG, Miller TP, Crowley JJ (1990). Chest irradiation (RT) *vs*. chest RT + chemotherapy + prophylactic brain RT in localized non small cell lung cancer: a southwest oncology group randomized study. Int J Radiat Oncol Biol Phys.

[25] Pöttgen C, Eberhardt W, Grannass A (2007). Prophylactic cranial irradiation in operable stage ⅢA non small-cell lung cancer treated with neoadjuvant chemoradiotherapy: results from a German multicenter randomized trial. J Clin Oncol.

[26] Gore EM, Bae K, Wong SJ (2011). Phase Ⅲ comparison of prophylactic cranial irradiation versus observation in patients with locally advanced non-small-cell lung cancer: primary analysis of radiation therapy oncology group study RTOG 0214. J Clin Oncol.

[b27] 27Wu YL. Prophylactic cranial irradiation (PCI) versus no PCI in non-small cell lung cancer after a pesponse to chemotherapy. (2008-09-02)[2012-08-15]. http://www.clinicaltrials.gov/ct2/show/NCT00745797?term=NCT00745797.

[b28] 28Komaki R. Combined modality treatment for resectable non-small cell superior sulcus tumors. (2011-09-28)[2012-08-15]. http://www.clinicaltrials.gov/ct2/show/NCT00984997?term=NCT00984997.

[29] Robnett TJ, Machtay M, Stevenson JP (2001). Factors affecting the risk of brain metastases after definitive chemoradiation for locally advanced non-small-cell lung carcinoma. J Clin Oncol.

[30] Dimitropoulos C, Hillas G, Nikolakopoulou S (2011). Prophylactic cranial irradiation in non-small cell lung cancer patients: who might be the candidates?. Cancer Manag Res.

[31] Keith B, Vincent M, Stitt L (2002). Subsets more likely to benefit from surgery or prophylactic cranial irradiation after chemoradiation for localized non-small cell lung cancer. Am J Clin Oncol.

[32] Bajard A, Westeel V, Dubiez A (2004). Multivariate analysis of factors predictive of brain metastases in localised non-small cell lung carcinoma. Lung Cancer.

[33] D'Amico TA, Aloia TA, Moore MB (2001). Predicting the sites of metastases from lung cancer using molecular biologic markers. Ann Thorac Surg.

[34] Bubb RS, Komaki R, Hachiya T (2002). Association of Ki-67, p53, and bcl-2 expression of the primary non-small-cell lung cancer lesion with brain *meta*static lesion. Int J Radiat Oncol Biol Phys.

[35] Gregor A, Cull A, Stephens RJ (1997). Prophylactic cranial irradiation is indicated following complete response to induction therapy in small cell lung cancer: results of a multicentre randomised trial. United Kingdom Coordinating Committee for Cancer Research (UKCCCR) and the European Organization for Research and Treatment of Cancer (EORTC). Eur J Cancer.

[36] Grosshans DR, Meyers CA, Allen PK (2008). Neurocognitive function in patients with small cell lung cancer: effect of prophylactic cranial irradiation. Cancer.

[37] Roman DD, Sperduto PW (1995). Neuropsychological effects of cranial radiation: current knowledge and future directions. Int J Radiat Oncol Biol Phys.

[38] Monje ML, Mizumatsu S, Fike JR (2002). Irradiation induces neural precursor-cell dysfunction. Nat Med.

[39] Gondi V, Tolakanahalli R, Mehta MP (2010). Hippocampal-sparing whole-brain radiotherapy: a " how-to" technique using helical tomotherapy and linear accelerator-based intensity-modulated radiotherapy. Int J Radiat Oncol Biol Phys.

[40] Tarnawski R, Michalecki L, Blamek S (2011). Feasibility of reducing the irradiation dose in regions of active neurogenesis for prophylactic cranial irradiation in patients with small-cell lung cancer. Neoplasma.

[b41] 41Brown PD. Memantine in preventing side effects in patients undergoing whole-brain radiation therapy for brain metastases from solid tumors. (2010-7-23)[2012-8-16]. http://www.clinicaltrials.gov/ct2/results?term=NCT00566852.

